# Effects of Improving Primary Health Care Workers’ Knowledge About Public Health Services in Rural China: A Comparative Study of Blended Learning and Pure E-Learning

**DOI:** 10.2196/jmir.6453

**Published:** 2017-05-01

**Authors:** Xingxin Zhan, Zhixia Zhang, Fang Sun, Qian Liu, Weijun Peng, Heng Zhang, Weirong Yan

**Affiliations:** ^1^ Department of Epidemiology and Biostatistics School of Public Health Tongji Medical College, Huazhong University of Science & Technology Wuhan China

**Keywords:** blended learning, e-learning, primary health care workers, public health services

## Abstract

**Background:**

Primary health care workers (PHCWs) are a major force in delivering basic public health services (BPHS) in rural China. It is necessary to take effective training approaches to improve PHCWs’ competency on BPHS. Both electronic learning (e-learning) and blended learning have been widely used in the health workers’ education. However, there is limited evidence on the effects of blended learning in comparison with pure e-learning.

**Objective:**

The aim of this study was to evaluate the effects of a blended-learning approach for rural PHCWs in improving their knowledge about BPHS as well as training satisfaction in comparison with a pure e-learning approach.

**Methods:**

The study was conducted among PHCWs in 6 rural counties of Hubei Province, China, between August 2013 and April 2014. Three counties were randomly allocated blended-learning courses (29 township centers or 612 PHCWs—the experimental group), and three counties were allocated pure e-learning courses (31 township centers or 625 PHCWs—the control group). Three course modules were administered for 5 weeks, with assessments at baseline and postcourse. Primary outcomes were score changes in courses’ knowledge. Secondary outcome was participant satisfaction (5-point Likert scale anchored between 1 [strongly agree] and 5 [strongly disagree]).

**Results:**

The experimental group had higher mean scores than the control group in knowledge achievement in three course modules: (1) module 1: 93.21 (95% CI 92.49-93.93) in experimental group versus 88.29 (95% CI 87.19-89.40) in the control group; adjusted difference, 4.92 (95% CI 2.61-7.24; *P*<.001); (2) module 2: 94.05 (95% CI 93.37-94.73) in the experimental group vs 90.22 (95% CI 89.12-91.31) in the control group; adjusted difference, 3.67 (95% CI 1.17-6.18; *P*=.004); (3) module 3: 93.88 (95% CI 93.08-94.68) in the experimental group versus 89.09 (95% CI 87.89-90.30) in control group; adjusted difference, 4.63 (95% CI 2.12-7.14; *P*<.001). The participants in the experimental learning group gave more positive responses with the four issues than control group participants: (1) the increase of interest in learning, 1.85 (95% CI 1.22-2.80; *P*=.003); (2) the increase of interaction with others, 1.77 (95% CI 1.20-2.60; *P*=.004); (3) the satisfaction with learning experience, 1.78 (95% CI 1.11-2.88; *P*=.02); and (4) achievement of learning objectives, 1.63 (95% CI 1.08-2.48; *P*=.02).

**Conclusions:**

Among PHCWs in rural China, a blended-learning approach to BPHS training could result in a higher knowledge achievement and satisfaction level compared with a pure e-learning approach. The findings of the study will contribute knowledge to improve the competency of PHCWs in similar settings.

## Introduction

### Background

In rural China, health services were delivered by a 3-tiered system consisting of county-level health care facilities, township hospitals, and village health clinics [1]. With the goal of providing affordable and equitable basic health care for all residents by 2020, the Chinese government launched a health care reform plan in April 2009. One of the main measures of this plan is the provision of a package of basic public health services (BPHS) for all residents. In 2015, the BPHS package included 13 kinds of services: health records management for residents; health education; vaccination; health management for children under 6 years of age; maternal health care; health care for the elderly; health care management of patients with hypertension, type 2 diabetes, severe mental illness, or tuberculosis (TB); reporting of infectious diseases and public health emergencies; health administrative oversight; and health management of Chinese traditional medicine [2]. Primary health care workers (PHCWs), especially those from village clinics and township hospitals, are at the bottom tier in terms of delivering most BPHS to rural residents.

Human resources is the crucial core of a health system, especially with regard to quantity and quality [3]. The competency of PHCWs can affect the delivery of BPHS in rural China, particularly the service quality [4]. Previous studies have revealed that most PHCWs, especially village doctors, have poor education and insufficient competency to provide high quality service [4,5]. One important strategy to improve the competency of PHCWs is training [6]. At present, the main training mode for PHCWs is the traditional face-to-face training [7], but its inflexibility, time constraints, travel costs, and limited training opportunities have negative effects on training [8,9]. Our previous qualitative study showed that the BPHS training was inadequate and ineffective in rural China [9]. Thus, there is a need for more effective solutions for training rural PHCWs on BPHS.

The increased popularity of the Internet and the growth of computer processing power during the past decade have provided opportunities for innovation and new approaches for training [10]. Alternatives to the traditional face-to-face training delivery, electronic learning (e-learning), and blended learning (a combination of e-learning and face-to-face learning) have been widely used in the health workers education [11-14]. Cook et al’s [15] systematic review reported that Internet-based learning had more positive effects when compared with no intervention in health professions, but more comparisons of different Internet-based interventions need to be conducted. To our knowledge, there is limited evidence on the effects of blended learning in comparison with pure e-learning [16-19]. In another systematic review in 2016, Liu et al [20] showed that blended learning is more effective or at least as effective as pure e-learning or pure traditional face-to-face learning among health professions and suggested that the more evaluation studies of blended learning, especially with e-learning should be conducted in future research.

### Aim of This Study

On the basis of the fact that most PHCWs in rural China need more effective training modes to improve their knowledge on BPHS, our study aimed to evaluate the effects of a blended-learning approach in improving BPHS knowledge among PHCWs in comparison with a pure e-learning approach.

## Methods

### Study Design, Setting, and Participants

 A comparative study was conducted in 3 cities (Yichang, Ezhou, and Xianning) in Hubei Province between August 2013 and April 2014. A multistage clustering sampling method was used to select participants in this study. In the first stage, according to their gross domestic product (GDP) rank in 2013 in Hubei Province, the cities of Yichang, Xianning, and Ezhou city were selected (low: Ezhou; medium: Xianning; high: Yichang). In the second stage, 2 counties with similar background characteristics in each city were selected; a total of 6 counties (Yiling and Zhijiang from Yichang city, Xianan and Chibi from Xianning city, and Huarong and Liangzihu District from Ezhou city) with 60 township centers were approached. In the third stage, the 2 counties in each city were randomly allocated to 2 groups, and therefore 3 counties, including 29 township centers were included in the blended-learning group (Zhijiang, Xianan, and Huarong counties; experimental intervention, 612 participants), and the other 3 counties, including 31 township centers, were in the pure e-learning group (Yiling, Chibi, and Liangzihu counties; control intervention, 625 participants). The selected counties in each city were at an average distance of more than 43 km. 

Included participants were PHCWs, either from township centers or village clinics within the administrative prefecture of each selected township, who are currently providing BPHS to rural residents. Exclusion criteria were refusal to provide informed consent, lack of space to attend the training, lack the basic computer skills, or lack of an Internet connection.

### Intervention and Data Collection

Three course modules were developed based on the BPHS contents [21]: Course module 1: health management of patients with hypertension; course module 2: health records management for residents; and course module 3: vaccination. Each course module consisted of 2 parts: theoretical learning and case studies. Both the theoretical and case materials were piloted in township centers and modified according to the feedback from interviews with experts and PHCWs in primary health institutions. The experimental and control groups had the same course materials. The public health services Web-based training platform based on Moodle was created for the study from August to October 2013 [22]. In addition, PHCWs outside the study area were invited to test the ease of use and stability of the training platform during the development period to ensure normal use of the platform. The experimental group received theoretical knowledge on the training platform and the cases delivered through the face-to-face method. In the control group, both theoretical knowledge and cases were delivered by the training platform.

All participants were enrolled in the study for an overall period of 5 weeks (1 week for trainees to familiarize themselves with training platform; 3 weeks for the theoretical learning; and 1 week for the case study). Before theoretical learning, all trainees could have access to the manual about training platform for 1 week and receive training or guidance for using the platform. I For the sake of consistency between the two groups, all study subjects were required to complete the theoretical learning of the three course modules first before starting the case studies. During the intervention implementation period, there was no regular meeting held at the county CDC (Center for Disease Control and Prevention) to reduce contaminations between the two intervention groups. Two facilitators were present during the training sessions of both groups for assistance and to answer questions. The details for the interventions are as follows.

#### Control Group

The pure e-learning group received Internet training on the training platform. Theoretical learning was presented in the format of Microsoft PowerPoint with 5-6 questions inserted into the slides, and a synchronous audio explanation was attached in each slide. Case studies consisted of 3 video sessions in which “real-world” examples or cases were delivered by a lecturer. Each case-study video, consisting of 4-5 cases, was about 30 min in length. All learning activities had to be completed independently at a self-paced rate. Two discussion forums were developed on the training platform, for the theoretical learning and case studies respectively. The discussion forum for the theoretical learning was set to separate groups, meaning only the same group learners could discuss and talk to each other, to reduce contaminations between the two intervention groups [23]. Another discussion forum for case studies was only available to pure e-learning trainees, and it encouraged them to discuss cases and ask questions.

#### Experimental Group

Participants in the blended group studied the same PowerPoint-based theoretical materials (available at the same training platform) during the same period. After that, participants received the handouts of all case-study materials for self-studying 4-5 days and attended 1-day (8-h) face-to-face case-study training. All cases were administered on the day by the same lecturers as in the videos in the meeting room at county CDC. PHCWs were encouraged to discuss the cases with educators and other physicians during the face-to-face training.

#### Assessments

 Assessment instruments consisted of two parts: the same pre- and posttest multiple-choice questions (MCQ) test in a different order to evaluate knowledge achievement, and a questionnaire to evaluate trainees’ satisfaction. Each trainee at the start answered the pretest questionnaire to gain access to the three training course modules for 4 weeks. After 4 weeks of learning, trainees were asked to complete the posttest MCQ for three course modules. Due to the various dropouts from each course module, there were different numbers of participants in each course training. After the completion of the three course modules, all participants were asked to fill out an online evaluation questionnaire during the following week.

#### MCQ Test to Knowledge

 A similar pre- and a posttest questionnaire was developed to measure trainees' knowledge achievement in each course module. A total of 3 knowledge MCQ tests were developed, consisting of a 10-item MCQ test in course module 1, a 15-item MCQ test in course module 2, and a 20-item MCQ test in course module 3. Both groups finished the precourse MCQ tests online within 60 min (each MCQ test under 20 min). Experimental group learners finished the post-course MCQ tests onsite, and control group learners finished them online—both within 60 minutes. All questions were scored as one point per correct response and zero points for an incorrect response. Scores were changed as a percentage of questions answered correctly.

#### Questionnaire to Evaluate Trainee’s Satisfaction With the Course and Training Methods

An additional 8-item questionnaire was administered to all participants to evaluate their experience with the courses and training methods on a 5-point Likert scale from 1 (strongly agree) to 5 (strongly disagree) after finishing the three course modules (both the theoretical learning and case studies). The questionnaire was piloted with 52 PHCWs and revised accordingly to ensure that the questions could be understood and answered well by all respondents. Cronbach alpha for the questionnaire was .975 according to the pilot study. Subjects who participated in the pilot test were excluded from the final analysis.

### Outcomes

The primary outcome was the difference between the control and experimental intervention group in knowledge achievement (measured by baseline and postcourse MCQ tests). The secondary outcome was the difference in trainees’ satisfaction with the courses and training methods between the control and experimental intervention groups (measured by an 8-item evaluation questionnaire)..

### Sample Size

The information regarding baseline knowledge, possible gains, and intracluster (intraclass) correlation coefficient was obtained from our pilot study work to calculate the sample size and power calculation. A total of 56 clusters (township centers) are needed to detect a knowledge gain of 5% in the experimental intervention compared with the control intervention using a 2-sided test, an alpha level of 5%, 80% power, assuming a standard deviation of 20, an intracluster (intraclass) correlation coefficient of .06, and expecting a mean cluster size of around 20.

### Statistical Analysis

Data was presented as mean with 95% CI. Responses to the baseline and postcourse assessments were scored, and comparisons between the 2 groups were made. The MCQ postscores were compared between the two groups using a multilevel linear mixed model, with intervention group, time of assessment (baseline or postcourse), and intervention × time interaction as fixed effects and township centers and participants as random effects. For evaluating participants’ satisfaction with the training modalities, the responses were computed on a 5-point Likert scale from 1 (strongly agree) to 5 (strongly disagree). Because very few participants chose scores of 3, 4, or 5, in the analysis, we combined responses with scores of 3, 4, and 5 into a single category “neutral or disagree.” Univariate logistic regression analysis was used to calculate the odds ratios (ORs) and 95% CI for comparing the difference between the two groups on each item of the questionnaire.

All comparisons were 2-sided and were considered statistically significant at *P*<.05. On the basis of Cohen guidelines [24], an overall between-group effect size for outcome variable was calculated by dividing the between-group difference by the within-cluster standard deviation, with effect sizes of 0.8 considered large, 0.5 considered medium, and 0.2 considered small. SAS version 9.1 (SAS Institute) was used for all analyses.

### Ethics and Consent

This study was approved by the Ethics Committee of Tongji Medical College, Huazhong University of Science and Technology. Written informed content was obtained from all study subjects before the study.

## Results

### Participants Characteristics and Study Participation

**Figure 1:**
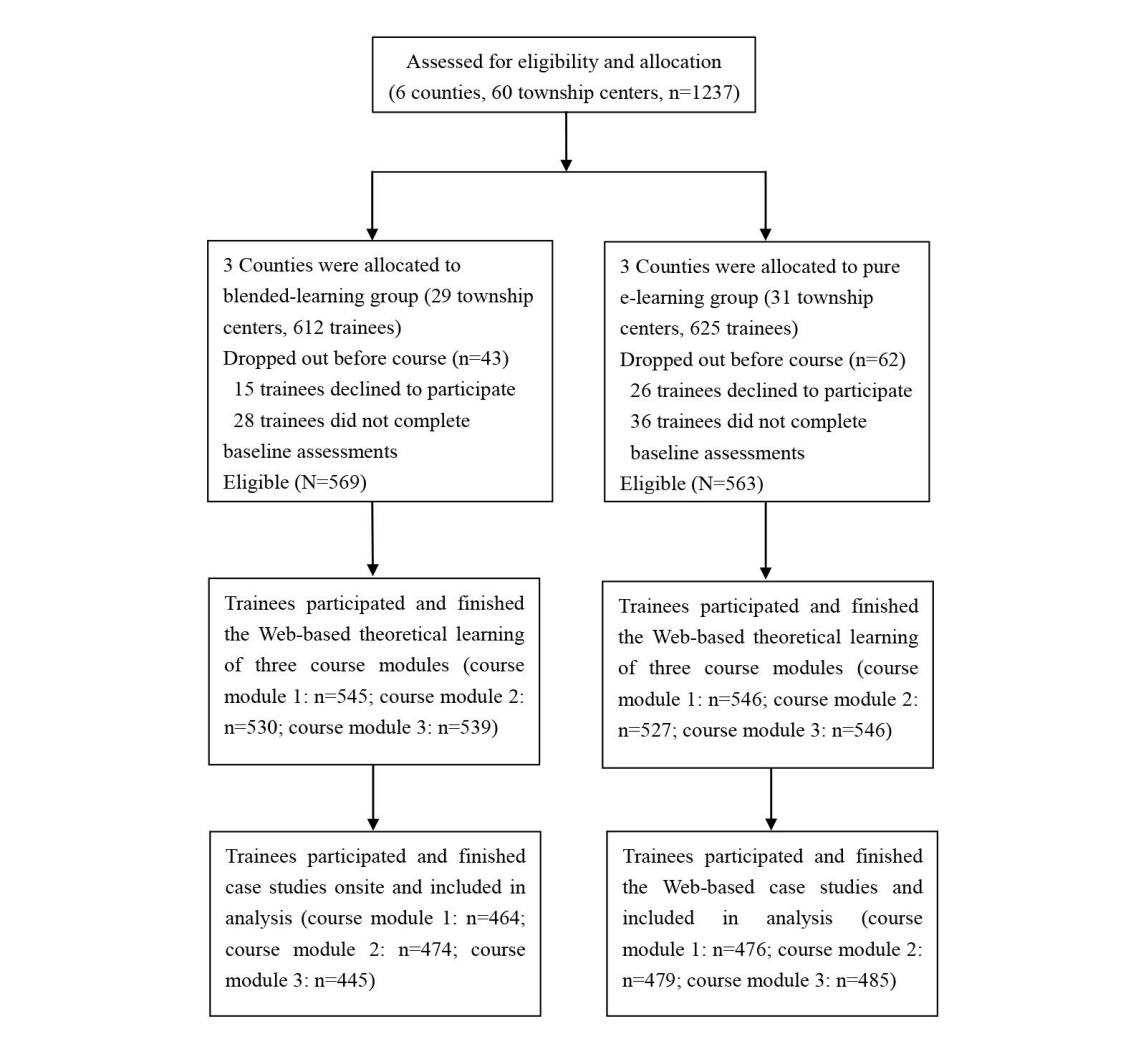
Study flow diagram.

 A total of 1237 PHCWs were recruited (Figure 1); 3 counties (Zhijiang, Xianan, and Huarong) with 29 township centers including 612 participants were assigned to the blended-learning group and 3 counties (Yiling, Chibi, and Liangzihu) with 31 towns including 625 participants were assigned to the pure e-learning group. A total of 43 participants in the blended-learning group and 62 in the pure e-learning group withdrew after the allocation due to refusal to participate or absence from baseline assessment. In total, 105 participants were lost to follow-up in course module 1; 95 in course module 2; 124 in course module 3 in the experimental group; and 87, 84, and 78 participants were lost to follow-up in course module 1, module 2, and module 3, respectively, in the control group.

Table 1 summarizes baseline characteristics of participants. Most participants had a technical secondary education background level or below and majored in western medicine. An analysis of baseline characteristics showed no statistically significant difference between the two groups.

**Table 1:** Demographic characteristics of the participants.

Variable		Blended-learning group (N=569)	Pure e-learning group (N=563)	DF	χ^2^/ *t*^c^	*P*
Mean age (SD^a^), years		41.67 (11.08)	41.98 (9.58)	1130	0.5	.59
**Age category, n (%)**						
	≤29 years	72 (12.7)	55 (9.8)	3	7.4	.06	
	30-39 years	184 (32.3)	182 (32.3)			
	40-49 years	165 (29.0)	200 (35.5)			
	≥50 years	148 (26.0)	126 (22.4)			
**Gender, n (%)**						
	Male	291 (51.1)	320 (56.8)	1	3.7	.06
	Female	278 (48.9)	243 (43.2)			
**Educational level, n (%)**						
	Technical secondary school or below^b^	453 (79.6)	435 (77.3)	2	1.1	.57
	Junior college	101 (17.8)	109 (19.4)			
	Undergraduate or above	15 (2.6)	19 (3.4)			
**Major, n (%)**							
	Western medicine	308 (54.9)	346 (61.5)	4	6.2	.18
	Nursing	129 (23.0)	113 (20.1)			
	Preventive medicine	52 (9.3)	40 (7.1)			
	Traditional Chinese medicine	29 (5.2)	31 (5.5)			
	Other	43 (7.7)	33 (5.9)			
						

^a^SD: standard deviation.

^b^Technical secondary school or below: illiterate or primary school, middle school, high school, or technical secondary school.

^c^ χ^2^/ *t*: *t* test was used to compare the “mean age (SD)” between two groups with χ^2^test comparing the differences between two groups in other variables such as “age category,” “gender,” “educational level,” and “major.”

### Knowledge Achievement

Baseline knowledge scores of the three course modules between experimental and control group were similar. After the interventions, there were more gains in the experimental group than in the control group: (1) Course module 1: postcourse mean, 93.21 (95% CI 92.49-93.93) in the experimental group versus 88.29 (95% CI 87.19-89.40) in the control group; adjusted mean difference, 4.92 (95% CI 2.61-7.24; *P*<.001). (2) Course module 2: postcourse mean, 94.05 (95% CI 93.37-94.73) in the experimental group versus 90.22 (95% CI 89.12-91.31) in the control group; adjusted mean difference, 3.67 (95% CI 1.17-6.18; *P*=.004). (3) Course module 3: postcourse mean, 93.88 (95% CI 93.08-94.68) in the experimental group versus 89.09 (95% CI 87.89-90.30) in the control group; adjusted mean difference, 4.63 (95% CI 2.12-7.14; *P*<.001). See Table 2. These gains represented moderate effect sizes for knowledge in these course modules (0.40, 0.34, and 0.40, respectively).

**Table 2:** Changes in knowledge using scores obtained with multiple-choice questions between blended-learning group and pure e-learning group.

Knowledge MCQ^a^ scores (%), mean (95% CI)	Blended-learning group N=464 for course module 1; N=474 for course module 2; N=445 for course module 3		Pure e-learning group N=476 for course module 1; N=479 for course module 2; N=485 for course module 3		Comparisons between two groups	
	Baseline	Postcourse	Baseline	Postcourse	Adjusted difference^b^, Mean (95% CI)	*P* value
Course module 1^c^	69.69 (68.10-71.27)	93.21 (92.49-93.93)	69.63 (68.16-71.1)	88.29 (87.19-89.40)	4.92 (2.61-7.24)	<.001
Course module 2^d^	71.20 (69.75-72.65)	94.05 (93.37-94.73)	72.71 (71.38-74.05)	90.22 (89.12-91.31)	3.67 (1.17-6.18)	.004
Course module 3^e^	74.12 (72.45-75.79)	93.88 (93.08-94.68)	73.85 (72.37-75.34)	89.09 (87.89-90.30)	4.63 (2.12-7.14)	<.001

^a^MCQ: multiple-choice questions.

^b^Adjusted difference is the mean difference between groups (intervention-control) adjusted for time of assessment and intervention × time interaction in a multilevel model with township center and participants as a random effect.

^c^Course module 1: health management of patients with hypertension.

^d^Course module 2: health records management for residents.

^e^Course module 3: vaccination.

### Trainee’s Satisfaction With the Interventions Methods

 A questionnaire response rate of 71.9% (409/569) was achieved in the blended-learning group compared with 80.3% (452/563) in the pure e-learning group. Trainees' subjective opinions toward the interventions were investigated, including training benefits (confidence increase, aim realization, and knowledge improvement), changes in learning interest, and satisfaction with the training mode and the interaction. A majority of PHCWs agreed that the contents were well relevant to their work (93.9% in experimental group vs 94.5% in control group, *P*=.70) and that they would like to try the training mode again (92.4% in experimental group vs 90.8% in control group, *P*=.37; Table 3). The blended-learning trainee was found to be more in agreement than the pure e-learning trainee due to the following four issues: (1) “Participation in the training had increased my interest in learning” OR 1.85 (95% CI 1.22-2.80; *P*=.003); (2) “Participation in the training increased the interaction with others” OR 1.77 (95% CI 1.20-2.60; *P*=.004); (3) “Overall, I was satisfied with learning experience” OR 1.78 (95% CI 1.11-2.88; *P*=.02); and (4) “I achieved the objectives of each course” OR 1.63 (95% CI 1.08-2.48; *P*=.02). Concerning other questions in the evaluation questionnaire, there were no significant differences found between the experimental and control groups (Table 3).

**Table 3:** Questionnaire evaluation of the training between the blended-learning and pure e-learning group.

Courses evaluation questions	Blended-learning group (N=409) n (%)^a^					Pure e-learning group (N=452) n (%)^a^					OR^b^ (95% CI)^c^	*P* value
	1	2	3	4	5	1	2	3	4	5		
1. The courses are relevant to the daily work.	157 (38.7)	224 (55.2)	15 (3.7)	5 (1.2)	5 (1.2)	169 (37.4)	258 (57.1)	24 (5.3)	1 (0.2)	0 (0)	0.89 (0.50-1.58)	.70
2. I achieved the objectives of each course.	141 (34.5)	228 (55.8)	30 (7.3)	9 (2.2)	1 (0.3)	139 (30.8)	245 (54.2)	64 (14.2)	4 (0.9)	0 (0)	1.63 (1.08-2.48)	.02
3. Participation in the training had increased my interest in learning.	153 (37.5)	216 (53.0)	32 (7.8)	5 (1.2)	3 (0.5)	147 (32.5)	231 (51.1)	61 (13.5)	12 (2.7)	1 (0.2)	1.85 (1.22-2.80)	.003
4. Participation in the training had increased my confidence.	150 (36.9)	211 (51.8)	31 (7.6)	14 (3.4)	1 (0.3)	159 (35.2)	230 (50.8)	43 (9.5)	19 (4.2)	1 (0.2)	1.27 (0.85-1.91)	.25
5. Participation in this training had improved my knowledge.	183 (45.5)	199 (49.5)	12 (3.0)	8 (2.0)	0 (0)	191 (42.3)	243 (53.8)	16 (3.5)	2 (0.4)	0 (0)	0.79 (0.42-1.52)	.48
6. Participation in the training increased the interaction with others.	155 (38.1)	206 (50.6)	35 (8.6)	11 (2.7)	0 (0)	144 (31.9)	226 (49.8)	70 (15.7)	12 (2.6)	0 (0)	1.77 (1.20-2.60)	.004
7. I would like to try the training mode again.	190 (46.6)	187 (45.8)	20 (4.9)	4 (1.0)	7 (1.7)	205 (45.4)	205 (45.4)	36 (8.0)	5 (1.1)	1 (0.2)	1.25 (0.77-2.02)	.37
8. Overall, I was satisfied with the training experience.	195 (48.3)	181 (44.8)	23 (5.7)	2 (0.5)	3 (0.7)	136 (30.1)	263 (58.2)	49 (10.8)	4 (0.9)	0 (0)	1.78 (1.11-2.88)	.02

^a^Responses to questions about the feedback on Web-based training platform were on a 5-point Likert scale, ranging from 1 (strongly agree) to 5 (strongly disagree).

^b^OR: odds ratio.

^c^Univariate logistic regression analysis was used to compare the differences between two groups (dependent variable as two categories with combining scores 1, 2 into one category and scores 3, 4, 5 to another category).

## Discussion

### Principal Findings

This study suggested that in rural China, a blended approach to BPHS training was more effective in improving knowledge than a pure e-learning approach. Trainees in blended-learning group expressed a higher satisfaction level about their learning experiences than pure e-learning trainees. Our study demonstrates the feasibility of applying Internet-related technology to PHCWs’ training on BPHS and explores the various training modes to improve the knowledge of PHCWs in rural China.

Currently, the inequalities in health care provision between urban and rural areas and the inequalities in the distribution of health workers remain serious problems in China [25-27]. Rural areas have both lower densities of health workers and less-educated workforce [6]. Achieving the equitable BPHS for all residents requires that every Chinese family in rural and urban areas has access to an appropriately trained and supported health worker. Our previous qualitative study showed that most PHCWs had insufficient knowledge on BPHS but had a positive attitude toward Web-based training approaches [9]. At present, e-learning has become an increasingly popular means to promote learning among health workers using online communications [15]. Blended learning, the combination of e-learning, and face-to-face instructor training, has also been presented as a promising approach for health education [10]. The differences between two novel methods include the different communication scenarios and perceived costs, with face-to-face scenarios having higher learners' costs [28]. In this study, we discussed the comparison results of blended and pure e-learning methods, focusing on two aspects: knowledge achievement and satisfaction level.

 Our study suggests that the blended-learning approach is more effective than pure e-learning in terms of knowledge achievement. This is supported by a recent meta-analysis of 56 studies finding that blended learning appears to be more effective than or at least as effective as e-learning [20]. Our findings are consistent with the previous research which showed that the combination of computer-assisted instruction and traditional classroom lecture yielded a significantly greater improvement in knowledge achievement of nursing students than when either strategy is used alone in the context of congenital heart disease [16]. Similarly, Llambí et al [19] reported that Uruguayan physicians who completed a blended-learning course on tobacco cessation achieved better test scores than those who attended pure online course. Furthermore, our quantitative results in this study also showed that blended learners expressed more positive ratings about goal achievement than online learners. A possible explanation may be that blended-learning approaches allow PHCWs to have face-to-face interactions and discussions within groups. A study conducted among pharmacy students emphasized the significance of face-to-face interactions in the blended-learning approach, which were more highly rated than online interactions [29]. Lack of face-to-face interaction was reported to be a challenge addressed in e-learning programs [30,31]. Previous studies suggested that lack of face-to-face interaction in the pure e-learning may contribute to professional isolation, a decrease in learning experience quality, and unsatisfactory learning outcomes [14,32,33]. According to constructivist learning theory, learning is a social activity, which is intimately associated with the connection with other human beings, teachers, peers, and so on [34]. The theory proposed that learners who have recognized the social aspect of learning and enhanced their interaction with others are more likely to have successful learning experiences [34]. This is further supported by our results showing that blended learners had increased interaction with others via the training than e-learners. Similarly, other studies indicated that blended-learning learners are less likely to experience feelings of isolation or reduced learning interest when compared with e-learners [20,35-37]. Consistent with the previous studies, blended learning achieved a greater learning interest in our study. The richness of blended experiences, including two forms of learning methods and allowing learners to have the face-to-face association and interaction with peers, might also promote learners’ learning interest.

In our study, we found that blended-learning trainees had a higher satisfaction level about their learning experiences than pure e-learning trainees. As for the case-based problem solving courses, social and collaborative learning experiences are important to help individuals in thinking, learning, and finding a solution for problems [34]. So and Brush [38] indicated that learner perceptions of collaborative learning were related to learning satisfaction, and learners with higher perceived levels of collaborative learning tended to be more satisfied with blended courses. Although participants in the pure e-learning group could communicate with others in the discussion forums on the BPHS Web-based platform, the asynchronous communication might not make trainees feel part of a learning community. Another study revealed that the learners in the online learning group claimed less learning support and more workload than learners in the blended-learning group with the explanation that learners in the Web-based learning group might lack a sense of presence or belonging [39]. Blended learning with various instructional methods, such as the mix of the face-to-face form of classroom training and Web-based technology, was the major factor in enhancing learner satisfaction [39,40].

Consideration of learning outcome alongside the devoted costs and resources was important for educators to effectively review the educational interventions [41]. Commonly, there are five basic cost-driving categories related to both blended-learning and pure e-learning approaches: labor costs, content development and acquisition, technology and infrastructure, operations costs, and learner-support services [42]. It was reported that developing a 100% online, media-rich, self-paced Web-based content was expensive and required multiple resources and skills [43]. Meanwhile, previous studies suggested that blended learning may potentially balance out and optimize the training program development and deployment cost and time by combining different delivery modes [43,44]. However, another study pointed out that not all blended learning would be cost-effective, and that the design of learning models around staff time was the determinant [45]. In addition, the resource support in the blended learning are involved in making different forms of resources (offline and online) available for learners as well as organizing them [43]. Thus, the cost-effective analysis of the blended versus pure e-learning approaches is necessary for educators to develop a more cost-effective mode, and we suggest that it needs to be conducted in the future.

Although blended learning shows positive learning outcomes and satisfaction level in the study, barriers to the implementation among PHCWs still exist. Possible barriers to blended learning include technical difficulties, such as interrupted or limited Internet connection, poor computer literacy, and hindrance in accessing learning resource material, as reported previously [11,46]. The lack of time for PHCWs to take part due to service load was another barrier because most rural PHCWs are responsible for delivering both BPHS and medical service to residents [5]. The barriers mentioned above could explain most dropouts in the blended-learning group in our study. Making suitable arrangement between work and training is critical for PHCWs to complete the training courses. On the other side, selecting the right blend between face-to-face and online learning is also important for the successful implementation of blended courses [47], which should take into account the job characteristics of health care workers.

### Strengths and Limitations

To our knowledge, this is the first study to compare the effectiveness of a blended-learning approach with a pure e-learning approach to BPHS training among rural PHCWs. The main strengths of this study include the relatively large sample size, and both subjective and objective evaluation methods applied for comparison. Furthermore, our study provided more evidence on the effects of blended learning in comparison with pure e-learning.

The study has five limitations. First, the dropouts in both groups were seen in our study. The dropout rates were similar in the blended group and pure e-learning group in course module 1 (18.5% vs 15.5%) and course module 2 (16.7% vs 14.9%). As for course module 3, we should caution that the dropout rate was differentially higher in the blended group (21.8%) than that in the pure e-learning group (13.9%). However, the background characteristics of participants who drop out in two groups in the course module 3 were similar. As well, there were no significant differences in the comparison of background characteristics between dropouts and non-dropouts. In addition, among dropouts who had completed the baseline assessment of course module 3, there was no baseline difference between those who completed (mean score 73.98 [SD 17.26]; n=930) and dropouts (mean score 72.29 [SD 23.75]; n=155) with difference, 1.69 (95% CI −1.43 to 4.81; *P*=.40). As there are some dropouts who had not completed the baseline assessment and their willingness had not been investigated, we still should caution the potential selective bias. Second, all subjects included in this study were from Central China, which limits its generalizability to other areas. Third, we compared the knowledge achievement between two training modes, but the effects of the courses on behavioral change or long-term educational outcomes were not evaluated and compared in this study. Fourth, the same knowledge questionnaires for pre- and posttest were used in the study, which might have introduced a subject sensitization bias. Fifth, the economic evaluation of two different training forms was not carried out in our study. To develop a more cost-effective training mode, the economic evaluation should be performed in a future study.

### Conclusions

In conclusion, blended approaches to BPHS training resulted in a better knowledge achievement and a higher satisfaction level than pure e-learning approaches among PHCWs in rural China. Using more effective training modes to improve PHCWs’ knowledge on BPHS can help enhance the PHCWs’ competency and accordingly improve the quality of health care in rural China in order to achieve health equity. To provide more rigorous evidence on the effects of blended learning in comparison with pure e-learning, more research is needed in the future.

## Abbreviations

BPHS: basic public health services
CDC: Center for Disease Control and Prevention
e-learning: electronic learning
GDP: gross domestic product
MCQ: multiple-choice questions
OR: odds ratio
PHCWs: primary health care workers
TB: tuberculosis
